# Modulation of p53 activity by IκBα: Evidence suggesting a common phylogeny between NF-κB and p53 transcription factors

**DOI:** 10.1186/1471-2172-6-12

**Published:** 2005-06-21

**Authors:** David H Dreyfus, Masayuki Nagasawa, Erwin W Gelfand, Lucy Y Ghoda

**Affiliations:** 1 Division of Basic Sciences, Department of Pediatrics, National Jewish Medical Research Center, Denver, CO 80262 USA; 2 Department of Pediatrics, Yale University School of Medicine, New Haven, CT, USA; 3 Departments of Pediatrics and Developmental Biology, Postgraduate School, Tokyo Medical and Dental University, Tokyo, Japan; 4 The Webb-Waring Institute for Cancer, Aging, and Antioxidant Research and the Department of Medicine, the University of Colorado at Denver and Health Sciences Center, Denver CO 80262 USA; To whom correspondence should be addressed at The Webb-Waring Institute, UCDHSC, Box C321, 4200 East Ninth Ave., Denver, CO 80262 USA

## Abstract

**Background:**

In this work we present evidence that the p53 tumor suppressor protein and NF-κB transcription factors could be related through common descent from a family of ancestral transcription factors regulating cellular proliferation and apoptosis. P53 is a homotetrameric transcription factor known to interact with the ankyrin protein 53BP2 (a fragment of the ASPP2 protein). NF-κB is also regulated by ankyrin proteins, the prototype of which is the IκB family. The DNA binding sequences of the two transcription factors are similar, sharing 8 out of 10 nucleotides. Interactions between the two proteins, both direct and indirect, have been noted previously and the two proteins play central roles in the control of proliferation and apoptosis.

**Results:**

Using previously published structure data, we noted a significant degree of structural alignment between p53 and NF-κB p65. We also determined that IκBα and p53 bind *in vitro *through a specific interaction in part involving the DNA binding region of p53, or a region proximal to it, and the amino terminus of IκBα independently or cooperatively with the ankyrin 3 domain of IκBα In cotransfection experiments, κBα could significantly inhibit the transcriptional activity of p53. Inhibition of p53-mediated transcription was increased by deletion of the ankyrin 2, 4, or 5 domains of IκBα Co-precipitation experiments using the stably transfected ankyrin 5 deletion mutant of κBα and endogenous wild-type p53 further support the hypothesis that p53 and IκBα can physically interact *in vivo*.

**Conclusion:**

The aggregate results obtained using bacterially produced IκBα and p53 as well as reticulocyte lysate produced proteins suggest a correlation between *in vitro *co-precipitation in at least one of the systems and *in vivo *p53 inhibitory activity. These observations argue for a mechanism involving direct binding of IκBα to p53 in the inhibition of p53 transcriptional activity, analogous to the inhibition of NF-κB by κBα and p53 by 53BP2/ASPP2. These data furthermore suggest a role for ankyrin proteins in the regulation of p53 activity. Taken together, the NFκB and p53 proteins share similarities in structure, DNA binding sites and binding and regulation by ankyrin proteins in support of our hypothesis that the two proteins share common descent from an ancestral transcriptional factor.

## Background

In this work we present evidence that the p53 tumor suppressor and NF-κB transcription factors could be related through common descent from a family of ancestral transcription factors regulating cellular proliferation and apoptosis. P53 and related proteins are transcription factors that regulate DNA repair and cellular apoptosis in response to stress and injury, notably those resulting in DNA damage [1-4]. Although it is a non-essential gene, loss of p53 function in humans through hereditary syndromes is associated with a markedly increased rate of malignancy. Furthermore, over 50% of malignancies have mutated p53 alleles [5]. These observations suggest p53 and related proteins function as a checkpoint for malignant transformation either by repairing DNA damage or by eliminating cells with irreparably damaged DNA [6-8]

P53 is a homotetrameric transcription factor that binds a consensus sequence 5' RRRRC(A/T)(T/A)GYYY-3' (where R indicates purine, A or G; and Y indicates pyrimidine, C or T)[9]. The consensus sequence is usually present as a dimer in p53 inducible gene promoters such as the p21^WAF1 ^protein regulating cell cycle progression [10]. P53 protein can be divided into three functional domains, the amino-terminal activation domain encompassing amino acids 1–43, the core sequence-specific DNA-binding domain (amino acids 100–300), and the multi-functional carboxy-terminal domain (amino acids 300–393) [11,12]. Point mutations in p53 identified in malignant cells are clustered around volutionarily conserved regions in the DNA binding region of p53 and simultaneously eliminate both sequence-specific DNA binding and transcriptional activity [12-14].

P53 is regulated on multiple levels including post-translationally by modifications such as acetylation, phosphorylation, protein degradation, and protein-protein interaction [15,16]. Phosphorylation of p53 induces conformational changes that alter interactions with regulatory proteins such as MDM2, which in turn can regulate p53 stability, and can also activate site-specific DNA binding activity [17-22]. Additional cellular proteins that bind to p53 include proteins of the general transcription machinery such as CBP/p300 [23,24]. CBP/p300 binding to p53 regulates acetylation and p53 transcriptional activation [25,26]. P53 is also regulated through association with ankyrin repeat proteins such as p53 binding protein 1 (53 BP1) and p53 binding protein 2 (53BP2, now known to be a fragment of apoptosis stimulating protein of p53 or ASPP2) [27] and gankyrin [28,29]. Thus, it is likely that p53 is modulated by association with and/or modification by a variety of regulatory proteins including kinases, transcription factors, and ankyrin-containing proteins. In addition, viral proteins also bind to and modify p53 and may contribute to malignant transformation of infected cells by viruses such as papilloma [30,31], cytomegalovirus [32], and Epstein-Barr virus (EBV)[33,34].

NF-κB transcription factors also play a central role in the control of apoptosis [35-37]. NF-κB transcription factors bind to the consensus sequence 5'-GGRNNYYCC-3' in the promoters of both cellular and viral genes [38,39]. The RelA/p65 subunit of NF-κB is regulated by the ankyrin repeat protein IκBα which masks the nuclear localization signal of the p65/p50 NF-κB heterodimer [40,41]. NF-κB-inducing signals are transmitted from the cell surface to the cytoplasm resulting in site-specific phosphorylation at two sites in the N-terminus of IκBα [42-45], conjugation of ubiquitin molecules to IκBα, and subsequent degradation of ubiquitinated IκBα by the 26S proteasome complex [46-49]. Degradation of IκBα in turn unmasks the nuclear localization signal of p50/p65 followed by translocation of the active transcription factor to the nucleus. Other NF-κB subunits including a homodimer of the p50 subunit also bind IκBα [50]. IκBα deficient animals while viable, die of uncontrolled inflammation in infancy, and mice overexpressing IκBα display an abnormal immunologic repertoire suggesting that a major physiologic role of IκBα is to limit immune and inflammatory responses through a feedback pathway [35,51,52].

Interactions between p53 and NF-κB have been noted, for example both factors compete for a binding site in the regulatory factor CBP/p300 [53]. Transfection of a constitutively active form of IκBα protein can block p53 dependent cell death [54-56] and p53 regulatory factors can modulate NF-κB pathways [57-59]. Not only are p53 and NF-κB transcription coregulated under a variety of physiological conditions, but similarity has been noted between the crystal structures of p53 and NF-κB p50 [14,60-62]. Both proteins contain a similarly sited zinc atom that coordinates site specific DNA binding and similar secondary and tertiary organization, but no primary amino acid similarity was noted between the two proteins. Furthermore, p65 has been shown to bind the ankyrin protein p53-binding protein 2 (53BP2). 53BP2 has been shown to be a fragment of a larger protein, ASPP2, that promotes the apoptotis-inducing effects of p53 [27]. P65 apparently inhibits p53-mediated apoptosis by binding to 53BP2/ASSP2 [63]. As with p53, NF-κB transcription is altered by viral pathogens [64-67].

In this work, we present evidence for the specific binding of the NF-κB inhibitor and ankyrin protein, IκBα, to p53. A physical interaction between p53 and IκBα was also reported by Chang [68], as well as Zhou, et al. [69], and was shown to be regulated by proapoptotic and growth suppressing stimuli. Our studies show the binding interaction to involve both ankyrin and non-ankyrin sequences in the IκBα protein, and the DNA binding core domain of p53. We demonstrate that transient expression of IκBα is associated with NF-κB independent decreases in p53 mediated transcription of a p53 reporter gene *in vivo*. These observations were made in Akata cells, an EBV-genome positive lymphoblastoid cell line originally derived from a Burkitts lymphoma. Akata cells lack endogenous functional p53 [33]. We propose that the binding of the ankyrin protein IκBα to p53 is based upon the similarity in molecular structure of NF-κB and p53.

## Results

### Similarity between p53 and NF-κB transcription element binding sites

The hemi-dyad DNA consensus binding sites of p53 and NF-κB transcription factors are intriguingly similar as has been noted previously by Foo, *et al*., [70]; for p53, the binding site sequence is 5'-RRRRC(A/T)(T/A)GYYY-3' and for NF-κB the binding site sequence is 5'- GGGRNNYYCC-3' where N, R, and Y indicate any nucleotide, purine (A or G), and pyrimidine (C or T), respectively. Two changes in the nucleotide sequence of the C(A/T)(A/T)G core (underscored, above) of a p53 binding site is sufficient to generate the RNNY core (underscored, above) of a NF-κB binding site while sequences flanking the core are conserved. Depending on the specific sequences, these binding sites potentially encode a hairpin structure that could promote these nucleotide substitutions. Under these circumstances, only one mutagenic event would be necessary, since the second nucleotide exchange could occur by excision-repair following a mutagenic event in the first site. Based on this observation, we hypothesized that both regulatory factors shared descent from a common ancestral transcription factor, a proto-p53/NF-κB. An alternative possibility is that two independent families of transcription factors, proto-p53 and proto-NF-κB, converged to independently recognize a similar recognition sequence.

**Figure 1:**
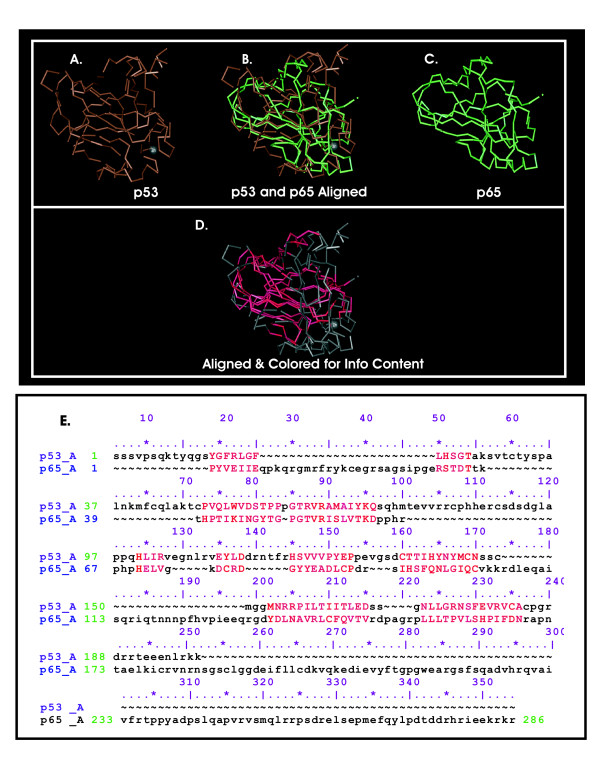
Alignment of p53 (PDB ID:1YCS; 1YCS_A d1) with p65 (PDB ID:1IKN; 1IKN_A d1). A. The structures of p53 molecule (taken from the crystal structure of p53 and p53 binding protein 2); B. The aligned structures of p53 and p65; C. The structure of the Rel homology domain of p65 (taken from the crystal structure of the p65/p50 heterodimer bound to IκBα); D. The aligned structures were colored according to information content based on a BLOSUM62 matrix to calculate conservation using the public domain program CN3D4.1 [70]. A spectrum of red to blue is used to denote the degree of conservation where red is the most conserved. E. The sequence alignment of p53 and p65 is depicted using the same coloring scheme as in the structure alignment in 1D.

If both p53 and NF-κB descended from a common ancestral protein, they might retain the ability to bind to common proteins or proteins of related structures in addition to binding similar DNA sequences. With independent descent converging upon a similar DNA binding sequence no such common interactions would be expected. In support of descent from a common ancestral protein, the crystal structure of NF-κB p65 aligns with that of p53 (Figure 1B and 1D, discussed below) a relationship that would not be expected unless the two proteins were related through phylogeny (descent) as well as ontogeny (function). Furthermore, numerous p53 binding proteins have in common the ankyrin repeat structure, a feature shared with the IκB family of NF-κB regulatory proteins.

### Structural alignment of p53 and p65 and ankyrin proteins, 53BP2 and IκBα

**Figure 2:**
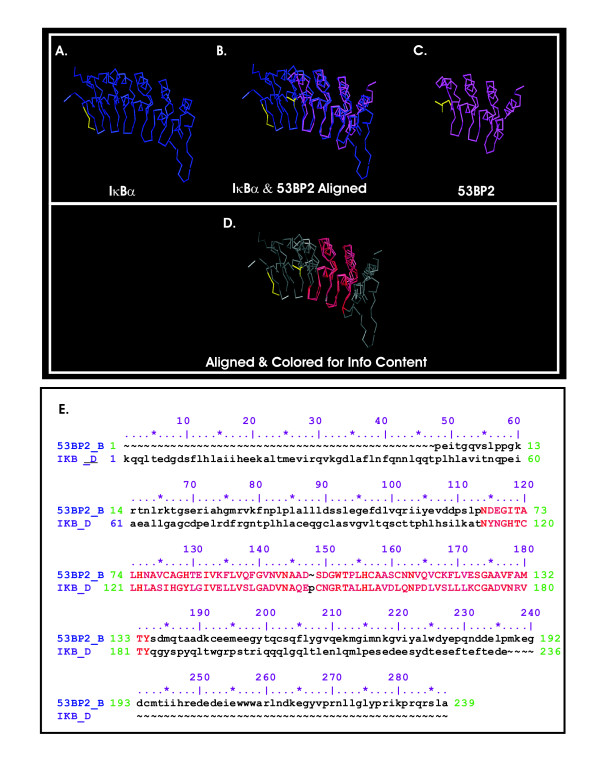
Alignment of IκBα (PDB ID:1IKN; 1IKN_D d1) with p53 binding protein 2 (PDB ID:1YCS; 1YCS_B d1). (A) The structure of IκBα (taken from the crystal structure of the p50/p65 heterodimer bound to IκBα); (B) The aligned structures of IκBα and 53BP2; (C) The structure of 53BP2 (taken from the crystal structure of p53 bound to 53BP2); (D) The aligned structure in 2B colored for conservation according to information content as described above; (E) The sequence alignment of IκBα and 53BP2 is depicted using the same coloring scheme as in the structure alignment in 2D. A four amino acid region near the N-termini of each structure is colored yellow as a reference point.

To test the hypothesis that p53 and p65 may share structural homology, the known crystal structures of both molecules were aligned using the program C3D4.1 [71]. P53 (Figure 1A) has previously been crystallized in association with the ankyrin protein 53BP2 (Figure 2C) [28]. Like IκBα (Figure 2A), 53BP2 is also an ankyrin protein and has been identified as a fragment of a larger protein known as ASPP2 (apoptosis stimulating protein of p53) [27]. The structure of p65 (Figure 1C) is taken from the crystal structure of the dimer comprised of p65 and p50 in association with IκBα [50]. P65 has also been crystallized as a homodimer in association with IκBβ [72].

We used the information content algorithm of C3D4.1 which uses a BLOSUM62 based matrix to calculate conservation between the two pairs of proteins: p53 and p65 (Figure 1E); and IκBα and 53BP2 (Figure 2E). The amino acids colored red in the aligned structures of 1D and 2D and the amino acid sequences of 1E and 2E depict regions of highest conservation while the grey areas are regions where there is no conservation. As shown by the regions of the two molecules colored red, we observed a surprising degree of structural alignment between the Rel homology domain of p65 and the p53 core domain, supposedly unrelated molecules (Figure 1D). The sequence alignment corresponding to the structures in 1D is depicted in 1E.

Using these same methods, we aligned the structures of the ankyrin proteins, IκBα [50] and 53BP2 (Figure 2D and 2E). The structure used for IκBα (Figure 2A) contains 71–280 of the wild-type protein. We omitted the SH3 domain of the 53BP2 structure (Figure 2C) since it is not relevant to this report. The IκBα protein contains six ankyrin motifs while the 53BP2 contains four. As expected, the two ankyrin proteins show a high degree of alignment. The fourth and 5^th ^ankyrins of IκBα are in very close alignment to the second and third ankyrins of 53BP2 (Figure 2D). The sequences corresponding to these structures is shown in Figure 2E.

### Specific binding between IκBα and p53 occurs *in vitro*

**Figure 3:**
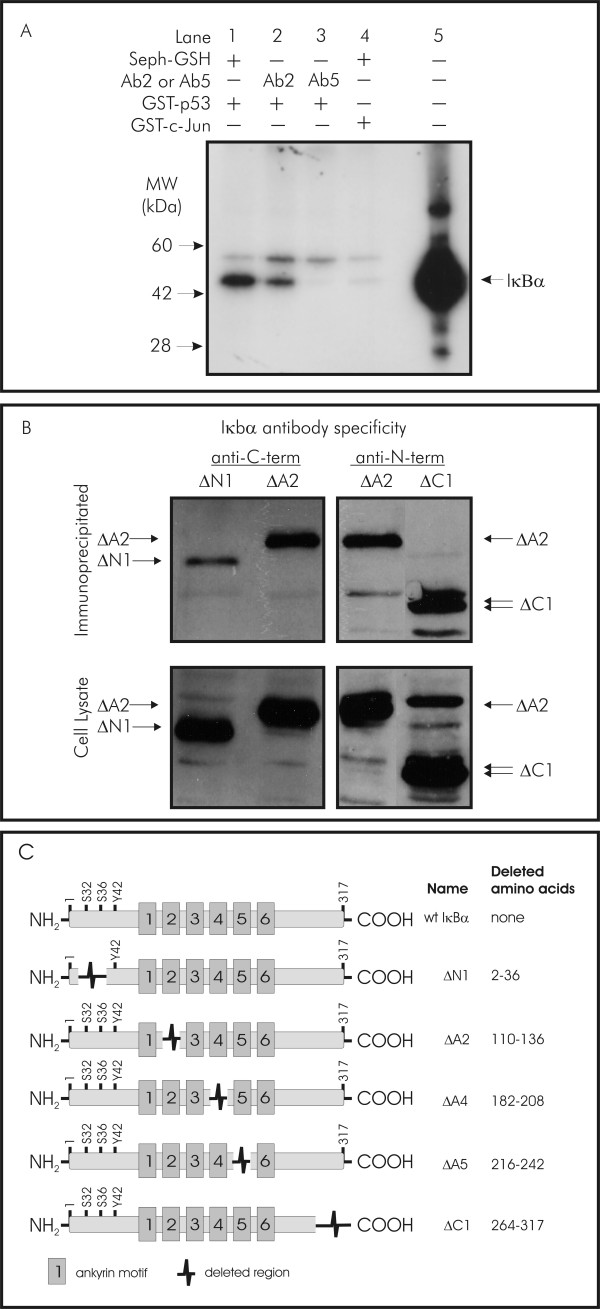
P53 and IκBα proteins co-precipitate *in vitro*. A: Purified bacterially produced IκBα protein co-precipitated specifically with p53. Purified IκBα protein was specifically labeled with [γ-^32^P]ATP using p90^rsk ^(lane 5). [^32^P]-labeled IκBα was incubated with purified p53 (GST-p53, lanes 1–3) or a control GST fusion protein (GST-c-Jun, lane 4). P53 was precipitated either by a glutathione binding tag on GST-p53 and glutathione Sepharose beads (Seph-GSH, lane 1) or a p53 specific antibody (Ab2, lane 2) and protein A/G Sephadex beads. IκBα protein (position indicated) was not precipitated by p53 specific Ab5 (lane 3) that does not recognize bacterially synthesized p53 protein, or by incubation with GST-c-Jun and precipitation with glutathione Sepharose beads (lane 4). Proteins were separated by PAGE and detected by autoradiography of [^32^P]-labeled protein. Electrophoresis of the input IκBα used in this experiment is also shown in lane 5. B: Relatively less ΔN1 protein than ΔC1 IκBα protein co-precipitated with GST-p53 from COS cell lysates. After expression of IκBα in COS cells, whole cell lysates were incubated with bacterially produced purified p53 (GST-p53). P53/ IκBα complexes were then precipitated with glutathione Sepharose beads and analyzed by PAGE/Western blotting. In the left panels, ΔN1 and ΔA2 proteins were detected using a rabbit polyclonal antisera directed at the C-terminus of IκBα, while in the right panels, ΔA2 and ΔC1 proteins were detected using a rabbit polyclonal antisera directed at the N-terminus of IκBα. C: Structures of wild-type IκBα and mutant constructs used in these studies.

Purified IκBα protein was specifically labelled *in vitro *with [^32^P] using p90^rsk ^which we have previously shown to quantitatively phosphorylate IκBα. [^32^P] -labeled IκBα was incubated with purified p53 (GST-p53) and precipitated either with glutathione/Sepharose (Seph-GSH) beads to bind the glutathione binding tag on GST-p53 or a p53-specific antibody (Ab2) and protein A/G beads. As shown in Figure 3, evidence of a specific association between purified bacterially-produced p53 and purified bacterially-produced wild-type IκBα was observed *in vitro *(Fig. 2A). The conformation-specific p53 antibody (Ab5) does not recognize bacterially produced p53 (unpublished observations), and Ab5 was used as a control for non-specific association between IκBα and antibody or Protein A/G beads. A c-Jun protein with a glutathione binding protein tag (GST-c-Jun) was used as a control for non-specific association between IκBα and glutathione beads. As shown, no association between control proteins or antibodies was evident.

### Relatively decreased *in vitro *binding between IκBα and p53 results from deletion of the N-terminus of IκBα

Initial experiments conducted with both p53 and IκBα made in bacterial cells supported the hypothesis that the p53 protein could bind to IκBα. Post-translational modifications of the proteins when expressed in eukaryotic cells could affect the interactions noted, particularly with respect to relative binding affinities of mutant proteins. Furthermore, bacterially produced protein may differ in conformation to those produced in mammalian hosts. Overexpression of IκBα alleles in COS cells was used to confirm that binding to p53 was evident with IκBα produced *in vivo *and also to map the p53 binding to sites within IκBα. Only some alleles of IκBα can be overexpressed in COS cells including IκBα deleted of N-terminal regulatory sequences, denoted ΔN1(missing amino acids 2–36), ΔA2 (missing amino acids 110–136), and ΔC1 deleted of the PEST-containing non-ankyrin C- terminus (amino acids 264–317). A schematic of deletion mutants of IκBα used is depicted in Figure 3C.

After expression of IκBα in COS cells, whole cell lysates were co-precipitated with bacterially produced purified GST-p53 (Figure 3B). These experiments confirmed that binding between the proteins was not related to production of an aberrant form of the IκBα protein in bacterial cells since alleles of IκBα protein produced in COS cells were enriched by incubation and co-precipitation with GST-p53. In representative experiments shown, relatively less ΔN1 protein protein co-precipitated with GST-p53 from COS cell lysates as compared to the proportions of ΔA2 or ΔC1 IκBα proteins associating with p53 suggesting that binding between the proteins at least in part required an intact N-terminal region of IκBα. Relative co-precipitation of the C-terminally deleted form of IκBα was comparable to that of wild-type protein (wild-type data not shown), suggesting that the amino acids 264–317 of IκBα was not important for binding to p53.

### The binding between IκBα and p53 is disrupted by an antibody to the DNA-binding region of p53

**Figure 4:**
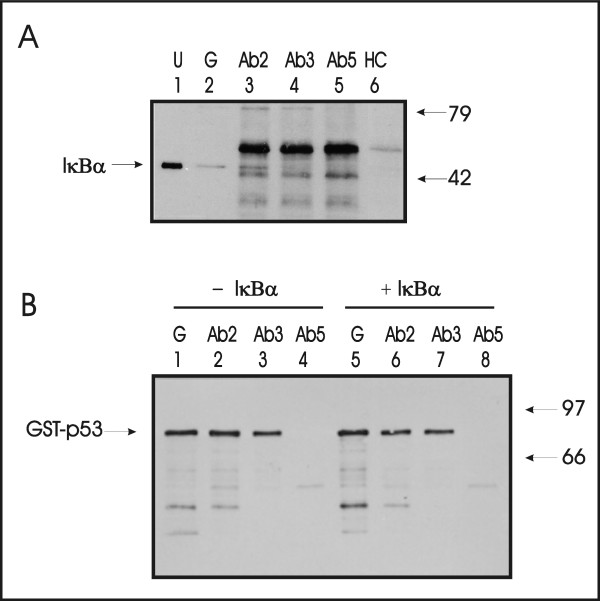
A monoclonal antibody recognizing an epitope in the DNA binding domain of p53 (Ab3) interferes with IκBα binding to p53. Purified p53 with a glutathione binding protein epitope tag (GST-p53) and purified IκBα protein were incubated together *in vitro*. A. P53 was precipitated either with glutathione Sepharose (denoted G, lane 2) or with p53-specific monoclonal antibodies Ab2 (lane 3), Ab3 (lane 4), or Ab5 (lane 5) and Sephadex protein A/G beads. Precipitated proteins were separated by SDS-PAGE and detected by Western blotting with a rabbit polyclonal antiserum directed against the N-terminus of IκBα. Mobility of IκBα protein is indicated (lane 1). This also represents the total input IκBα. Similar quantities of murine immunoglobulin heavy chain (HC, lane 6) were precipitated by protein A/G beads and served as the negative control. B. GST-p53 was quantitatively precipitated in the absence (- IκBα) or presence (+ IκBα) of IκBα by glutathione Sepharose (denoted G, lanes 1,5), Ab2 (lanes 2,6), and Ab3 (lanes 3,7), but not Ab5 (lanes 4,8), as detected with a rabbit polyclonal p53 antiserum. This blot is essentially identical to that shown in panel A but for the antibody used in the Western blotting step. Since Ab5 did not precipitate GST-p53, it served as a negative control for non-specific association between IκBα protein and either antibody or protein A/G beads.

Experiments detailed above confirmed that a specific binding association occurs between IκBα and p53 *in vitro*. Further experiments were designed to approximate the region on p53 responsible for IκBαbinding by use of epitope-specific monoclonal antibodies. Glutathione-sepharose beads (G) were used to precipitate p53 (Figure 4A, lane 1, and 4B, lanes 1 and 5) and were found to co-precipitate IκBα. A monoclonal antibody directed at amino acids 46–55 in the amino terminus of p53 (Ab2) recognized purified bacterially produced p53 (4B, lanes 2 and 6) and also co-precipitated IκBα with p53 (4A, lane 2). In contrast, a different p53-specific monoclonal antibody directed at amino acids 212–217 within the DNA binding core of p53 (Ab3), while able to recognize and precipitate bacterially produced p53 protein (4B, lanes 3 and 7), did not co-precipitate IκBa (4A, lane 4). These observations suggested that the binding site between purified IκBα and p53 in part coincided with the Ab3 epitope, a region which is within the p53 DNA binding core. Alternatively, the antibody could hinder or disrupt IκBα binding to regions near the recognized. epitope or alter the structure of p53 into a conformation unfavorable for IκBα binding.

### Overexpression of wild-type IκBα modulates p53 dependent transcription of a synthetic p53 reporter gene *in vivo*

*In vitro *studies supported the hypothesis that a direct physical association between p53 and IκBα can occur but they did not provide evidence that such interactions occur *in vivo*. We hypothesized that transient overexpression of IκBα in cells with co-transfected transcriptionally active p53 might reveal effects of IκBα upon p53 mediated transcription. Using forced overexpression of both proteins from identical viral CMV promoter elements rather than endogenous promoters would eliminate confounding transcriptional effects of IκBα upon transcription of p53 or *vice versa*. Any effect of NF-κB upon CMV would be normalized as well.

**Figure 5:**
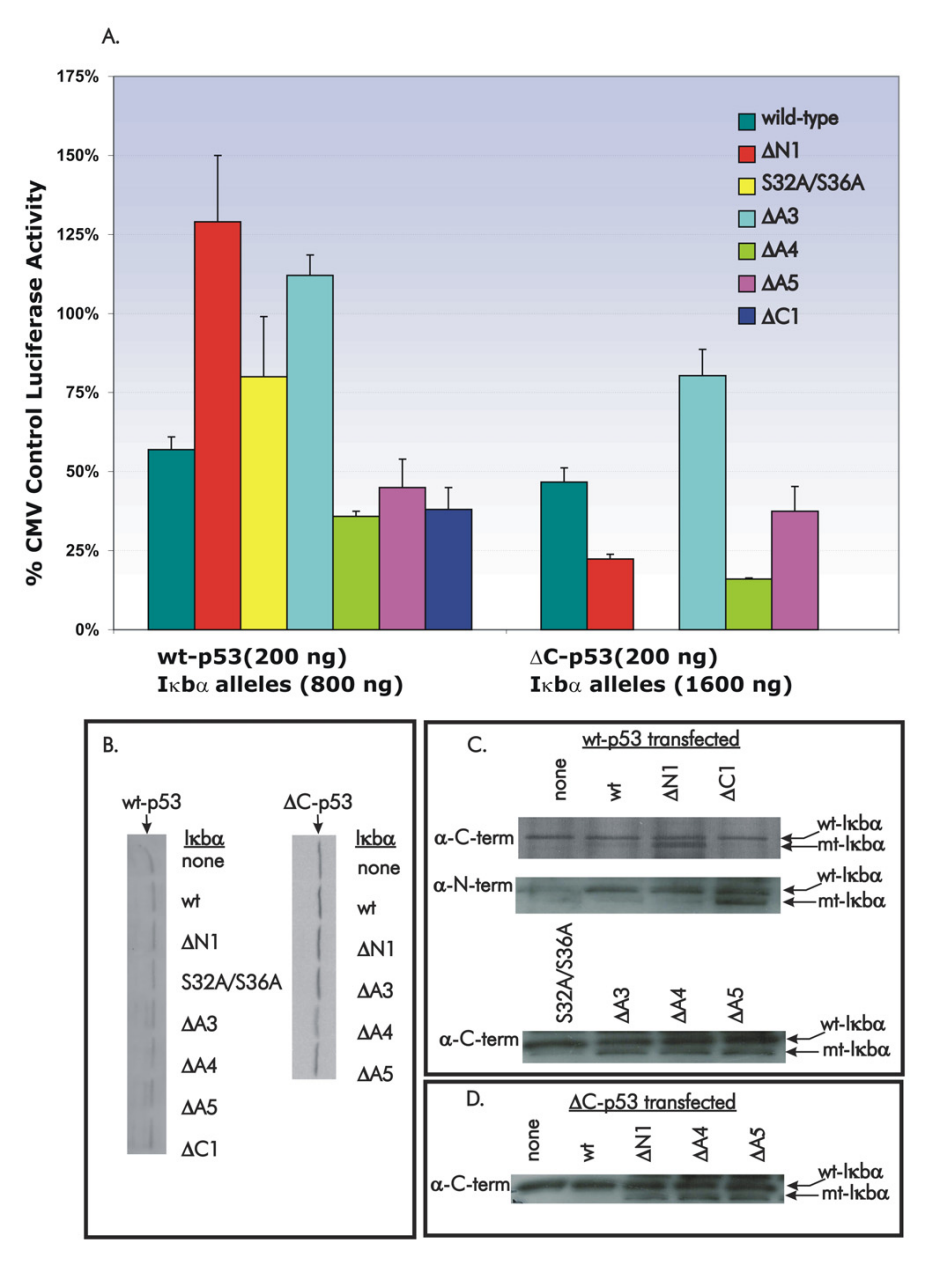
Transient transfection of IκBα alleles specifically blocks p53 transcription in EBV-positive Akata cells. A. 200 ng of a p53-dependent reporter plasmid encoding 13 copies of the p53 response element driving a firefly luciferase (pG(13)Py/Luc) were cotransfected into 2 × 10^5 ^cells with 1 ng of an SV40 promoter driven *Renilla *luciferase (pRLSV40) and 200 ng of a CMV-promoter driven wild-type p53 cDNA construct (wt-p53, denoted below the x-axis) or a C-terminally deleted transcriptionally active p53 (ΔC-p53, denoted below the x-axis). The effect of 800 ng of CMV-promoter driven IκBα alleles or a control construct that did not contain DNA encoding IκBα (to which all data were normalized) were used to determine the effect of IκBα on wild-type p53-mediated transcription of pG(13)Py/Luc while 1600 ng of CMV-promoter driven IκBα alleles (or control) were used in experiments where the ΔC-p53 allele was used. Immunoblotting controls for wt-p53, ΔC-p53 and IκBα alleles. B: Western blot analysis of wt-p53 and ΔC-p53 in the presence of transfected IκBα alleles. Extracts were derived from Akata cells as described for Figure 5A. None indicates transfection of empty CMV vector containing no IκBα allele. C: Western blot analysis for IκBα in the presence of transfected wt-p53. A subset of extracts were analyzed both with C-term and N-term directed IκBα antibodies. Endogenous IκBα is detected by these methods. D: Western blot analysis of IκBα in the presence of ΔC-p53. ΔA3 was analyzed on a separate gel due to space limitations and showed a band intensity similar to that of ΔA4 and ΔA5 (data not shown).

Akata cells were used because these B-lymphoblastoid cells are known to lack endogenous p53. Neither the wild-type nor a mutant protein is expressed that could complicate interpretation of results. P53 transiently transfected into these cells using dextran sulfate is transcriptionally active. As a control for non-specific effects of transfected plasmid DNA, effects of IκBα expression plasmids were compared to co-transfected plasmid containing an identical CMV promoter element (pCMV). Results shown were also normalized to expression of co-transfected pRL-SV40, a plasmid expressing a second form of luciferase as a control for cell viability and transfection efficiency. We found that wild-type IκBα was associated with decreased p53-dependent transcription of the p53 reporter gene pG(13)PyLuc in Akata cells (Figure 5). Increasing the relative ratio of transfected expression plasmids between IκBα and p53 from 1:1 (equivalent to 200 ng IκBα plasmid transfected) to 10:1 (equivalent to 2000 ng IκBα plasmid transfected) demonstrated a dose response effect that varied with different IκBα alleles. Immunoblotting for wt-p53, ΔC-p53, and IκBα alleles show relatively equivalent protein levels for these proteins in these extracts (Figure 5B–D).

### Allele specific modulation of p53 transcription by IκBα are independent of the effects of the alleles on NF-κB transcription *in vivo*

The wild-type IκBα protein is subject to signal-dependent phosphorylation and subsequent degradation leading to release of inhibition of NF-κB. Deletion (*e.g*. as in the ΔN allele deleting amino acids 2–36) or mutation of the phosphorylation sites (S32 and S36) in the amino terminus of IκBα leads to a stable mutant that constitutively represses NF-κB transcription. Cotransfection at a 1 to 1 ratio of ΔN1 to p53 expression plasmid (200 ng each of p53 and IκBα plasmid transfected) had no significant effects upon p53 transcription (data not shown). Increasing the relative ratio of ΔN1 expression plasmid to p53 showed that at higher ratios, ΔN1 could significantly decrease p53-dependent transcription (Figure 5A, inhibition of the constitutively active ΔC-p53 is shown at 1600 ng ΔN1 plasmid. Similar results were obtained when the ratio of ΔN1 to wild-type p53 was increased to 8:1, data not shown). However, at the lower ratio of 4:1 shown for the wild-type p53 allele, in Figure 5A, there was little inhibition of transcriptional activity by ΔN1. The relatively poor inhibitory activity of ΔN1 towards p53 is consistent with its relatively poor binding affinity for p53 as shown in Figure 3B and described above.

The finding that the ΔN1 allele is only partially competent to inhibit p53 is consistent with our previous observation that deletion of the N-terminus of IκBα reduced its physical affinity for p53 (Figure 3). While the ΔN1 allele is a super-repressor of NF-κB, the ΔA5 allele does not repress NF-κB, and thus this allele could be used to ask whether p53 repression and NF-κB inhibition are separable characteristics of the IκBα molecule. Remarkably, ΔA5, which cannot repress NF-κB transcription, had very pronounced inhibitory effects upon transcription by p53 (Figure 5A), comparable to that of wild-type.

### Mapping of interactions between IκBα and p53 *in vivo *to the ankyrin 3 domain of IκBα

Ankyrin regions in IκBα, shared with other members of the IκB gene family are the sites of binding to NF-κB transcription factor family members. Further experiments were performed to define the effects of deletion of additional ankyrin domains of IκBα upon p53 transcription in Akata cells. In these experiments, shown in Figure 5, IκBα plasmids were transfected at 800 ng (at a ratio of 4:1 relative to p53 plasmid). Deletion of either the second (ΔA2, amino acids 110–136, not shown) or fourth ankyrin domains (ΔA4, amino acids 182–208) had the most potent inhibitory effects upon p53 transcription. Deletion of amino acids 264–317 in the carboxyl terminus (ΔC1), distal to the 6^th ^ankyrin domian, were similarly inhibitory. This region retains the 6^th ^ankyrin domain but deletes a potentially regulatory acidic PEST region. Deletion of the ankyrin 1 domain of IκBα was not evaluated in this work.

In multiple experiments with wild-type p53, deletion of the ankyrin 3 (ΔA3) domain of IκBα (missing amino acids 143–169) resulted in a loss of inhibitory activity towards p53 transcription. Thus ΔA3 acts as a null allele for both p53 and NF-κB transcription *in vivo*, while other ankyrin deletion alleles of IκBα (ΔA2, ΔA4, ΔA5) act as null alleles for NF-κB but gain of function with respect to p53. Thus, our data are consistent with the hypothesis that the ankyrin 3 and N-terminus of IκBα are cooperatively or independently involved in repressing p53. We cannot, however, rule out an alternative possibility, that the deletion of the ankyrin 3 (ΔA3) of IκBα resulted in a protein with high turnover in Akata cells.

### Relative effects of alleles of IκBα are conserved with constitutively active p53 and require transcriptionally active p53

P53 transcription is regulated in part by phosphorylation of the carboxyl terminus of the protein. Phosphorylation induces a conformational change in p53 so that an auto-inhibitory region of the carboxyl terminus no longer inhibits DNA binding [73-75]. To determine whether the effects of IκBα upon p53 transcription required the auto-inhibitory carboxyl terminus of p53, experiments were repeated with a truncated form of p53 containing the first 353 amino acids (Figure 5A, ΔC-p53) together with an 8-fold excess of IκBα plasmid (1600 ng). As expected, these experiments demonstrated increased p53 transcriptional activity measured as p53 -dependent transcription of pG(13)PY/Luc in Akata cells at the same plasmid concentrations compared to experiments using wild-type p53. Co-transfection of a fixed concentration of a plasmid expressing ΔC-p53 protein (200 ng), and IκBα alleles (1600 ng) demonstrated that the carboxyl terminus of p53 was not required for the association between p53-dependent transcription and IκBα expression. As in experiments with wild-type p53, deletion of the ankyrin 4 (ΔA4) domain of IκBα was associated with greater decreases in p53-dependent transcription than other alleles of IκBα. A transcriptionally inactive p53 mutated in the p53 DNA binding core (pC53-CSX3, V193A) gave very low levels of luciferase activity that were not altered by co-transfection of any IκBα alleles (data not shown). Thus, the effects of IκBα upon p53 transcription in Akata cells were found to be entirely dependent upon the presence of co-transfected transcriptionally active p53 but independent of the carboxyl-terminus regulatory sequences of p53.

### Allele specific association between IκBα and p53 synthesized in a rabbit reticulocyte lysate (RRL) system correspond to allele specific modulation of transcription *in vivo*

**Figure 6:**
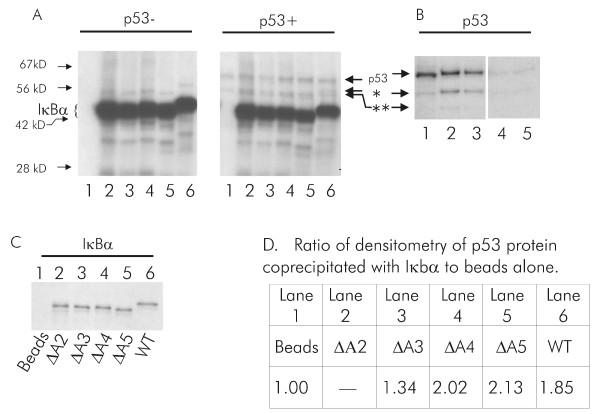
Rabbit reticulocyte lysate (RRL) produced p53 and IκBα proteins interact *in vitro*. A. Wild-type IκBα and mutant proteins were synthesized in rabbit reticulocyte lysates (lane 1, RRL no RNA; lane 2, ΔA2; lane 3, ΔA3; lane 4, ΔA4; lane 5, ΔA5; and lane 6, wild-type) and precipitated with a rabbit polyclonal antiserum against the C-terminus of IκBα using protein A/G sephadex beads either in the absence (p53-) or in the presence (p53+) of RRL synthesized, [^35^S]-labeled, p53. Equal volumes of RRL were used in all cases. For p53- reactions, RRL programmed with empty vector was incubated with [^35^S]-labeled methionine. A control reticulocyte lysate without IκBα protein template (lane 1) was included in these experiments. ΔA2 (lane 2), ΔA3 (lane 3), ΔA4 (lane 4) ΔA5 (lane 5) and wild-type (lane 6) IκBα proteins were co-incubated with p53, precipitated and analzyed by SDS-PAGE followed by radiography. B. Wild-type p53 protein translated in RRL (lane 1) produced both putative full-length p53 protein (denoted p53, mobility approximately 55 kD) and also at least two less-than-full length translation products (denoted * and **; lanes 1–3). Putative full-length and less-than full-length p53 translation products were precipitated by p53-specific monoclonal antibodies and protein A/G sephadex beads (lane 2, Ab1 recognizing the carboxyl terminus of p53; lane 3, Ab2 recognizing the amino terminus of p53). Less-than full-length proteins were more readily precipitated by Ab1 than Ab2. Control precipitation with IgG2A immunoglobulin (lane 4) or protein G/A beads (lane 5) did not precipitate p53. C. IκBα proteins were precipitated in similar quantities by IκBα antiserum. A shorter exposure of the gel than in panel A is shown. D. Densitometry of p53 protein precipitated in association with various IκBα alleles. Data shows significant precipitation of p53 with ΔA4, ΔA5 and wild-type, expressed as a ratio of label precipitating with IκBα allele to beads alone. Data was calculated by first normalizing to the background in each lane followed by calculating the ratio of label precipitating with IκBα to a corresponding area in the beads alone lane.

Some alleles of IκBα including ΔA3 and ΔA4 were not stable in COS cells (unpublished observations). Therefore, COS cells could not be used to determine the correlation between IκBα and p53 binding *in vitro *(Figure 2B) and transcriptional effects in Akata cells (Figure 5). Thus, to overcome this technical problem, p53 and IκBα proteins were both separately synthesized in a rabbit reticulocyte lysate system (Figure 6). IκBα synthesized in the reticulocyte lysate system is functionally active in its effects upon NF-κB transcription factors and ubiquitin-dependent degradation. Likewise, but in contrast to bacterially synthesized p53, p53 synthesized in RRL system is functionally active as a site-specific DNA-binding protein and like IκBα is degraded *in vitro *by a ubiquitin-dependent pathway. Experiments were performed to determine whether RRL produced p53 that could interact with IκBα alleles likewise translated *in vitro *in RRL.

Coupled transcription/translation of a plasmid encoding wild-type p53 in RRL (lane 1, Figure 6B) resulted in several template-specific products that could be precipitated from lysates with p53-specific monoclonal antibodies (lanes 2 and 3, Figure 6B). These products appeared to be a mixture of full length and less-than full-length p53 proteins resulting from internal initiation sites in the p53 template. Negative control precipitation with IgG2A immunoglobulin (lane 4) or protein G/A beads (lane 5) resulted in undetectable levels of p53 recovery.

IκBα was precipitated with a polyclonal antiserum directed at the IκBα C-terminus in the absence (p53-) or presence of p53 (p53+) protein after both proteins were synthesized in the reticulocyte lysate system (Figure 6A). Deletion of the carboxyl terminus of IκBα did not interfere with binding between GST-p53 and IκBα synthesized in COS cells (Figure 2), suggesting that binding of antibody directed at the IκBα carboxyl terminus would not interfere with binding between p53 and IκBα. Visual inspection and gel densitometry of these co-precipitation experiments confirmed a specific association between wild-type IκBα and p53. The association of ΔA3 IκBα allele to p53 was significantly reduced (Figure 6A, p53+, lane 3) as determined by gel densitometry (Figure 6D, p < 0.05) relative to the association between the wild-type IκBα, ΔA4, and ΔA5 alleles of IκBα to p53. Gel densitometry was not sensitive enough to determine whether small differences in the binding of other IκBα alleles to p53 were significant. In the absence of labeled IκBα, bands corresponding to p53 were still evident (5A, lane 1, p53+) suggesting either non-specific binding or co-precipitation with endogenous unlabeled IκBα. There are small quantities of immuno-detectable IκBα in certain preparations of RRL (L. Ghoda, unpublished observations). Similar results were obtained in experiments in which an antibody against p53 (Ab2) were used to co-precipitate p53 and IκBα alleles (unpublished observations). Binding was also evident between IκBα and p53-related proteins smaller in size than full-length p53 although these interactions could not be reliably quantitated due to variable co-migrating protein species in the relevant portions of the gel.

### *In vivo *binding of ΔA5-IκBα to wild-type p53

**Figure 7:**
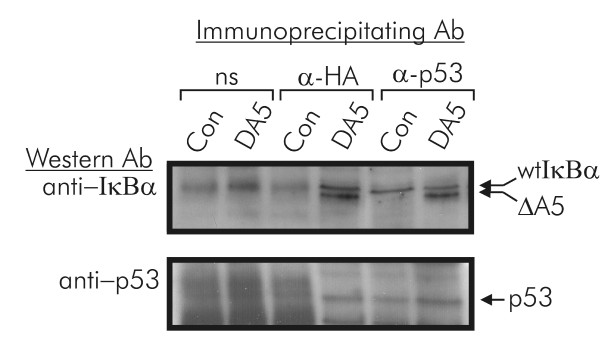
P53 associates with ΔA5-IκBα in UV-irradiated melanoma cells. A2085 cells stably transfected with the parent vector (Con) or ΔA5-IκBα allele (DA5) were irradiated for 30" with UVB and harvested 4 hrs later. The extracts were subjected to immunoprecipitation with non specific rabbit serum (ns), polyclonal rabbit anti-HA antibody (α-HA), or mouse monoclonal anti-p53 Ab2 antibody (α-p53) and probed by western analysis with mouse anti-IκBα antibody directed at the C-terminus of IκBα (top panel) or the Ab3 anti-p53 monoclonal antibody (bottom panel). Experiments were performed on stably transfected, pooled transfectants of controls (Con) or ΔA5-IκBα (DA5).

The melanoma cell line A2085 expressing endogenous wild-type p53 was stably transfected with the hemagluttinin (HA)-tagged ΔA5 allele of IκBα [76]. The ΔA5 allele was chosen because it has no effect on NF-κB activity but is one of the more potent alleles of IκBα in modulating p53-dependent transcription. Several independent stable clones as well as pooled transfectants were expanded and used for experiments for which results of the pooled transfectants (DA5) are shown here (Figure 7). Results were qualitatively similar with pooled *vs *cells expanded from single colonies. Pooled transformants resulting from antibiotic selection of vector transfected cells were used as controls (Con). Association of ΔA5-IκBα with p53 was evident when UV-irradiated cell extracts were immunoprecipated using anti-p53 antibody and the immunoprecipitates probed with anti-IκBα antibody (top panel). Likewise, association between transfected IκBα and p53 are evident when immunoprecipitates generated using anti-HA were probed with anti-p53 (bottom row). Immunoprecipitation with normal rabbit serum (ns) precipitated little or no IκBα or p53. Association between endogenous wild-type IκBα and p53 are also evident as seen by the upper band detectable with anti-IκBα western blotting in Con and DA5 lanes when extracts are precipitated with anti-p53. When extracts of irradiated cells are immunoprecipitated with anti-HA, and immunoblotted with anti-IκBα, Both endogenous and ΔA5 are evident. This is likely due to the precipitation of wt- IκBα with the p53/ ΔA5-IκBα complex since p53 exists as a tetramer.

## Discussion

In this work we determined that IκBα and p53 bind *in vitro *through a specific interaction in part involving the DNA binding region of p53, or a region proximal to it, and the amino terminus of IκBα independently or cooperatively with the ankyrin 3 domain of IκBα. A physical interaction between p53 and IκBα has been noted by two other groups [68,69]. Generally, our data corroborate these previously reported interactions and provide further evidence for direct transcriptional modulation of p53 by IκBα. There are some notable differences in our observations from that of others, in particular, the binding site on IκBα was reported as existing in the non-ankyrin C-terminus by Chang [68] in an yeast two-hybrid system using a deletion construct missing amino acids 244-317. This construct in fact deletes the 6^th ^ankyrin domain of IκBα, located at amino acids 244 – 263, retained in our C-terminal deletion construct (Δ264–317) which also retains p53 binding and regulation. When the data are taken *in toto*, it can be inferred that in fact the 6^th ^ankyrin domain, retained in our construct but deleted in Chang's, may in fact contain another p53 contact point, the loss of which were observed by Chang but not by us.

We also found that deletion of the ankyrin 2, 4, or 5 domains of IκBα increased the inhibitory effect of IκBα on p53-dependent transcription in Akata cells. We speculate that the compacting of the structure by elimination of an ankyrin domain may promote better binding of the protein to p53 as it may convert IκBα to a structure closer in size to that of 53BP2 which only contains four ankyrins. Mutations in the ankyrin regions of IκBα have been characterized regarding their effects upon NF-κB-mediated transcription as well as nuclear translocation, with most deletions of ankyrins including deletion of ankyrin 5 leading to a null phenotype (confirmed in part in this work in Akata cells) with respect to NF-κB transcription. Nevertheless, over-expression of ΔA5-IκBα was more effective than wild-type at decreasing p53 transcription at all but the lowest concentrations examined. Thus, it was evident that over-expression of some alleles of IκBα could influence p53 transcription independently of their effects upon transcription by NF-κB. Deletion of the ankyrin 3 region of IκBα eliminated detectable interactions between IκBα and p53 *in vivo *and *in vitro *suggesting a critical role of the ankyrin 3 region in a specific *in vivo *interaction with p53. The combined results obtained using bacterially produced IκBα and p53 (Figure 2) as well as RRL produced proteins (Figure 6) suggest a correlation between *in vitro *co-precipitation in at least one of the systems and in *vivo *p53 inhibitory activity. These observations argue for a mechanism involving direct binding of IκBα to p53 in the inhibition of p53 transcriptional activity.

**Figure 8:**
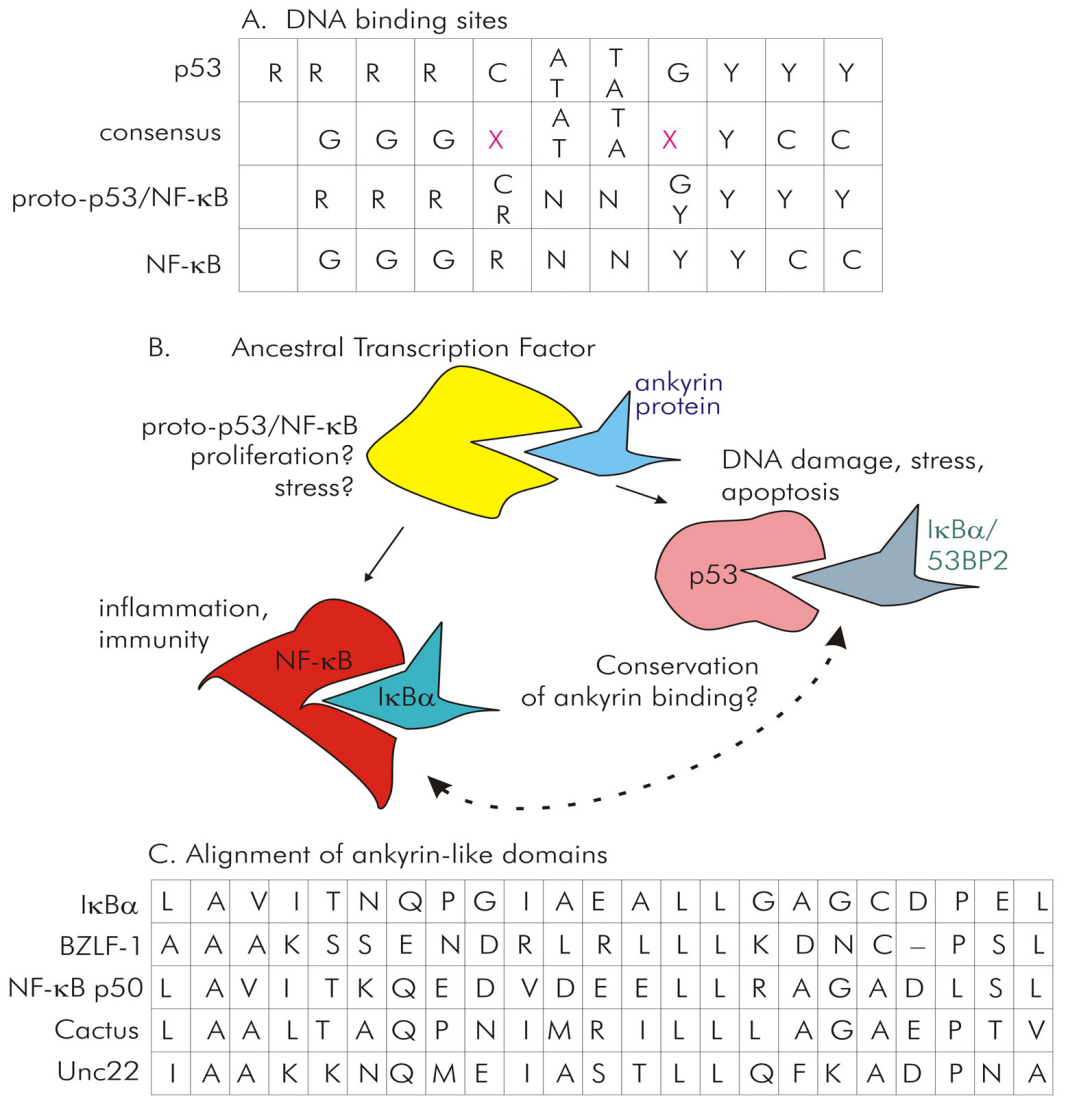
A model for molecular evolution of p53 and NF-κB from a common ancestral transcription factor, proto-p53/ NF-κB. A. P53 (first row) and NF-κB (fourth row) DNA binding sites share eight out of ten nucleotides as shown by the sequence depicted in the second row (consensus). R represents purine, A or G; Y represents pyrimidine, C or T. The red X denotes nucleotides where there is no match. The predicted DNA binding site sequence of the ancestral proto-p53/NF-κB is shown in the third row. B. An ancestral transcription factor proto-p53/ NF-κB, also regulated by an ankyrin protein, with a DNA binding site shown in A, above, could have been the precursor to both p53 and NF-κB. C. An ankyrin-like region in EBV protein BZLF-1 (ZEBRA) is shown in alignment with ankyrin motifs in IκBα, NF-κB p50, as well as invertebrate ankyrin like regulatory repeats from *Drosophila *Cactus and *C. elegans *Unc22.

Our interpretation of these observations is that binding between IκBα and p53 occurs primarily between the p53 DNA-binding core or a region proximal to it, and the ankyrin 3 and N-terminal regions of IκBα. Binding results in decreased p53-dependent transcription when both IκBα and p53 are overexpressed *in vivo*. These results are consistent with those published by Zhou, *et al*. [69] where induction of p53 regulatable genes such as p21 and Mdm2 in response to doxorubicin was abrogated by expression of a degradation-resistant form of IκBα. These interactions are strikingly similar to the known interactions between IκBα and its physiologic ligand, NF-κB. We suggest that these interactions are a result of a conserved relationship, *i.e*., that of a common descent, of p65 and p53 from a primordial transcription factor which we term proto p53/NF-κB (Figure 8B). Not only do the two transcription factors bind ankyrin proteins but both NF-κB and p53 have similar DNA binding sites (Figure 8A). Furthermore, both factors bind a viral protein encoded by the EBV BZLF-1 open reading frame (also known as ZEBRA) and can transcription can be modulated by this viral protein when overexpressed in lymphoblastoid cell lines [64,77]. As shown in Figure 8C, we found that a cryptic ankyrin like region is present in the dimerization region of BZLF-1, coincident with the region required for binding to both p65 and p53. With the exception of ankyrin domains, BZLF-1 and IκBα are otherwise not similar in sequence, structure, or function.

Thus, ankyrin repeat domains appear to be key points of interaction between diverse proteins in the NF-κB and p53 superfamilies. A similar conclusion is suggested by the crystal structure of a complex between p53 and 53BP2 [28]. 53BP2 is a 1002 amino acid protein containing four ankyrin repeats that has been found to be a fragment of an even larger protein known as ASPP2. Residues TYSD located in the 4^th ^ankyrin repeat of 53BP2/ASPP2 (Figure 2E, 133 TYsd) binds to the L2 loop of p53 immediately after the zinc ligand site in p53, while the non-ankyrin, SH3 domain of 53BP2 also contribute to the binding interactions. These four residues in 53BP2/ASPP2 are exactly aligned with an equivalent block in the sixth ankyrin repeat of IκBα (Figure 2E, 181 TYqq). It is interesting to note that this corresponds to the 6^th ^ankyrin domain predicted to be a contact point for p53 as outlined above. Paradoxically, 53BP2/ASPP2 is exclusively cytoplasmic thus it may only inhibit and sequester p53 in the cytoplasm. IκBα thus may fall into a paradigm of endogenous and viral p53 regulatory proteins where ankyrin and non-ankyrin domains contribute to p53 binding and transcriptional modulation. Other ankyrin proteins that bind to either NF-κB or p53 could also bind and potentially modulate each other.

## Conclusion

In conclusion, modulation of p53 transcription by IκBα and related host and viral proteins could play a role in the regulation of p53-dependent apoptosis *in vivo*. Both p53 and IκBα are members of multi-gene families, and both protein families regulate apoptosis. Previously, a cooperative relationship between p53-dependent apoptosis and NF-κB activation had been reported [59]. Our observations may represent the converse of this situation where inactivation of NF-κB *(i.e*. as a result of elevated IκBα levels) results in inhibition of p53 transcriptional activity. Alternatively, a situation where accumulation of IκBα free of NF-κB, as a result of phosphorylation of p65 by RSK1 and ensuing dissociation of NF-κB from IκBα, could result in the regulation of p53 by IκBα [78]. Another setting where our observations may play a significant physiological role is during herpesvirus infection. The Epstein-Barr nuclear antigen (EBNA1) contains a region enriched in gly-ala repeats, and this repeat sequence inhibits the 26S proteasome leading to inhibition of peptide presentation by the MHC Class I restricted pathway [79]. EBNA1 is expressed during viral latency, a condition where it is beneficial for the virus to inhibit apoptosis. Proteasome inhibition will lead to inhibition of all cellular protein degradation and the impact would be the greatest on those proteins with extremely short half-lives such as p53 and IκBα. Thus, the accumulation of IκBα under these circumstances may down regulate the activity of p53.

Decreased p53-dependent transcription could limit the ability of p53 to trigger cellular apoptosis in the presence of inflammation since levels of targets of NF-κB, including IκBα, are up-regulated. If, in fact, IκBα inhibits or dampens p53 function, cells could continue to proliferate and differentiate in the presence of an inflammatory event even if there is DNA damage or other cellular injury that would normally activate p53-dependent cell cycle arrest and apoptosis. Thus, a combination of inflammation and cellular damage could increase malignant transformation of cells since the threshold for p53 levels to trigger apoptosis may be elevated. It is notable that we know little about what thresholds regulate p53 function – in particular, what determines whether a cell repairs DNA and resumes transit through the cell cycle or dies. A molecule such as IκBα may be involved in influencing threshold-dependent cell fate decisions. The observation that truncated p53 proteins may bind to IκBα raises the possibility that short p53-related peptides could alter the binding between IκBα and p53, in turn altering a putative regulatory relationship between these proteins *in vivo*. Modulation of this regulatory interaction by pharmacologic or other means could potentially alter the balance between cellular proliferation and cell cycle arrest/apoptosis in the context of inflammation.

## Methods

### *In vitro *precipitation of p53 and IκBα

Bacterially produced 6xHis-tagged IκBα(His-IκBα) was labeled *in vitro *with ^32^P using [γ-^32^P]ATP and *Xenopus laevis *p90^rsk ^(a gift of Dr. James Maller, Howard Hughes Memorial Institute and UCHSC, Denver CO) [80] and detected with polyclonal rabbit antiserum specific to the N- or C-terminus of IκBα as described (a gift of Dr. Warner Greene, the Gladstone Institute for Virology & Immunology, UCSF, San Francisco, CA) GST-p53 produced from a pGEX construct in bacteris was incubated with hexahistidine-tagged IκBα purified as described in [44] in IP buffer buffer containing 25 mM Hepes, pH 7.5, 75 mM KCl, 2.5 mM MgCl_2_, 0.1 mM EDTA, 0.15% NP-40, and 1 mM DTT. Protein complexes were purified using either glutathione conjugated Sepharose beads to bind GST (Pharmacia) for GST-p53 or Protein A/G conjugated Sephadex beads (Oncogene Science, CA). Proteins were separated by polyacrylamide gel electrophoresis (PAGE) on either 10% or 12% gels which were dried and autoradiographed on photographic film (Fuji) for detection of radiolabeled protein or transferred to supported nitrocellulose or PVDF membrane and probed with antibodies using the Renaissance system for western blotting (NEN, Boston, MA). p53 specific monoclonal antibodies Ab1, Ab2, Ab3, Ab5, and Ab6 were obtained from Oncogene Science [81].

### Expression of IκBα alleles in cells

COS cells and Akata cells were transiently transfected using Superfectin (Qiagen, Chatworth, CA) with expression vectors for IκBα alleles in an expression plasmid regulated by a CMV promoter (pCMV5) obtained from Dr. S.C. Sun (Penn State, Hershey, PA). COS cells were lysed in IP buffer (see above) and incubated with p53-pGEX. Overexpression and coprecipitation of IκBα proteins in COS cells was complicated by the apparent instability of some IκBα alleles (ΔA3, ΔA4) these cells and lack of an association between other alleles (ΔA5) and p53-pGEX (unpublished observations). Following PAGE, ΔN1 IκBα protein was detected using a rabbit polyclonal antisera directed at the C-terminus of IκBα, while ΔC1 IκBα protein was detected using a rabbit polyclonal antisera directed at the N-terminus of IκBα in these experiments (antibodies to N- and C-termini of IκBα were a gift of Dr. W.C. Greene, the Gladstone Institute for Virology and Immunology and the University of California, San Francisco, CA).

### Transient transfection of Akata cells

Akata cells were obtained from Dr. J. F. Jones (National Jewish Medical Research Center, Denver, CO). The identity of Akata cells was confirmed by expression of EBV lytic gene products following ligation of surface IgG (data not shown). Cells were cultured using standard conditions in RPMI medium (GIBCO-BRL) supplemented with 10% fetal calf serum, penicillin/streptomycin (100 U/ml) and L-glutamine (2 mM). As previously reported, endogenous p53 protein was not detected in Akata cells by Western blotting with a rabbit polyclonal antisera directed at the entire p53 coding sequence (Santa Cruz Biologicals, Santa Cruz, CA) [21,81].

Akata cells were transiently transfected with plasmids using dextran sulfate (Pharmacia). p53 protein transiently expressed by plasmids transfected into Akata cells could be detected in nuclear extracts of transfected cells (data not shown). Levels of transfected wild-type IκBα protein were not detected by Western blotting above a high background of endogenous IκBα proteins in Akata cells, although expression of IκBα protein could be inferred by effects on transcription of NF-κB reporter genes. Akata were grown to a density of 1 × 10^6 ^cells/ml and 1 ml of cells for each experiment were transfected with Dextran. After transfection, cells were incubated in fresh culture medium for 24 hours prior to determination of luciferase activity.

### Luciferase assay

A luciferase reporter gene regulated by 13 tandem copies of a p53 response element, denoted pG(13)PyLuc, or p21 promoter reporter, denoted WWP/Luc, were transfected into Akata cells in experiments at concentrations indicated. As noted in the text the relative effects of different alleles of IκBα upon p53 dependent luciferase expression varied with the relative ratios of expression vector for IκBα to expression vector for p53. CMV-promoter driven p53 expression plasmids encoding wild-type p53 (pC53-SN3) and a DNA binding mutated p53 (pC53-CSX3 V193A) were obtained from Dr. B. Vogelstein, (Johns Hopkins University, Baltimore, MD). An expression plasmid encoding a carboxyl-terminus deleted p53 (pCEP4-353) was obtained from Dr. J. Pietenpol, Vanderbilt Univ., Memphis, TN). Plasmids were prepared using either the Promega (Madison, WI) or Qiagen (Chatsworth, CA) procedures with similar results.

A plasmid encoding a second form of luciferase (Renilla luciferase) driven by an SV40 promoter was used as an internal control for transfection efficiency and cell viability (pRL-SV40, Promega, Madison WI). Similar results were obtained without pRL transfection by normalization of luciferase activity to total cellular protein. Luciferase activity was measured using the Stop and Glo assay (Promega) and an Analytical Luminescence Laboratory luminometer (San Diego, CA). Each data point shown from luciferase assay experiments represents pooled data from at least three independent experiments. Standard error and significant differences (p < 0.05, indicated with an asterick, *) were as determined by the students t-test for multiple comparisons of data points using statistical software (SAS Institute Inc, Cary, NC).

### Reticulocyte lysate expression and immunoprecipitation

For reticulocyte lysate expression and immunoprecipitation studies, IκBα expression vectors were digested with XbaI/HindIII and ligated into the XbaI/HindIII site of pBS KS(Stratagene) to place the open reading frames downstream of a T7 RNA polymerase promoter element. These IκBα constructs lack epitope tags. Vectors encoding p53 wild-type (SN3) and DNA binding mutant p53 protein (pBKS-273) for reticulocyte lysate expression were obtained from Dr. B. Vogelstein. ^35^S-Cysteine/methionine labelled IκBα and p53 proteins were produced in the TNT coupled transcription/translation system (Promega) using T7 RNA polymerase for wild-type p53 and IκBα and T3 for mutant p53. 10 μl of reticulocyte lysates, as indicated, were incubated in a total volume of 100 μl IP buffer for 2 hours at 4°C. Antibodies were added and incubated for an additional 1 hour, and proteins were precipitated with Protein G plus/A Sephadex (Oncogene Science). Beads were washed once with 500 μl IP buffer and proteins denatured in Laemmli sample buffer and separated on 10 or 12% PAGE gels dried and visualized by autoradiography. Despite possibly confounding variables due to the presence of the p65 (RelA) subunit of NF-κB and possibly other related factors in reticulocyte lysates (unpublished observations), a correlation was evident between the quantity of p53 coprecipitated with IκBα and the relative effect of individual alleles upon *in vivo *p53 transcription in Akata cells. In particular, there was a lack of a detectable interaction between the ankyrin 3 deletion mutant of IκBα and p53 *in vivo *or *in vitro*.

## Abbreviations used

53BP2, p53 binding protein 2

ASPP2, apoptosis stimulating factor of p53 -2

EBNA, Epstein-Barr nuclear antigen

EBV, Epstein-Barr virus

## Authors' contributions

DHD and MN contributed equally to this work. Richard Ruhlen is acknowledged for his technical assistance.
